# Overexpression of Peroxisome-Localized *GmABCA7* Promotes Seed Germination in *Arabidopsis thaliana*

**DOI:** 10.3390/ijms23042389

**Published:** 2022-02-21

**Authors:** Jianchun Li, Zaihui Peng, Yan Liu, Meirong Lang, Yaohui Chen, Huihong Wang, Yingshuang Li, Banruo Shi, Weipeng Huang, Li Han, Yifeng Ma, Yu Zhang, Bangjun Wang

**Affiliations:** 1 Chongqing Key Laboratory of Plant Resource Conservation and Germplasm Innovation, Key Laboratory of Eco-Environments in Three Gorges Reservoir Region (Ministry of Education), College of Life Sciences, Southwest University, Chongqing 400715, China; ybxyli@163.com (J.L.); zaihpeng@foxmail.com (Z.P.); 13271925385@163.com (Y.L.); lmr1902379220@163.com (M.L.); zwjh2017@126.com (Y.C.); huihongwang@163.com (H.W.); yingshuangli2020@163.com (Y.L.); 13320353872@163.com (B.S.); huangweipengswu@163.com (W.H.); hanlimango@163.com (L.H.); ma_yifeng8803@126.com (Y.M.); 2 College of Food Science, Southwest University, Chongqing 400715, China; y000063@swu.edu.cn

**Keywords:** peroxisome, β-oxidation, seed germination, GmABCA7, soybean

## Abstract

Peroxisome is one of the important organelles for intracellular lipid metabolism in plant cells and β-oxidation of fatty acids in peroxisomes provides the energy for oil-containing seed germination. In this study, we identified an ATP-binding cassette (ABC) transporter gene, *GmABCA7* from soybean, which is highly expressed in the different developmental stages of seeds. Transient expression of *GmABCA7* in tobacco epidermal cells showed that GmABCA7 was specifically localized at the peroxisomes. Overexpression of *GmABCA7* in Arabidopsis does not change seed phenotypes, or the overall levels of lipid, protein and sugar stored in the seeds; however, the transgenic seeds produced more gluconeogenic pathway precursors such as succinate and malate and germinated earlier compared to the wild type seeds. These results suggest that GmABCA7 may affect the β-oxidation of fatty acids and play an important role in seed germination.

## 1. Introduction

Soybean (*Glycine max*) is a major oilseed crop worldwide that is also rich in proteins. The oil in soybean seeds is mainly in the form of triacylglycerol (TAG), providing a carbon source and energy for seed germination and seedling establishment. During germination and seedling development, TAG breaks down into fatty acids, which are then converted to succinate in peroxisomes through fatty acid β-oxidation and glyoxylate cycles, and it ultimately produces sucrose through the gluconeogenic pathway [1]. Thus, plant peroxisomes are the central organelles for storage lipid degradation and are critical for seed germination. Fatty acids can enter a peroxisome with the help of a variety of transporters like the ATP-binding cassette (ABC) transporter AtABCD1 [2].

ABC transporters are an important class of transmembrane proteins that are widely present in organisms, and their structures and functions are highly conserved [3]. The Arabidopsis genome encodes more than 100 ABC transporters [4,5]. Based on the transmembrane domains (TMDs) and the nucleotide-binding folds (NBFs), all plant ABC proteins are divided into eight major subfamilies, namely, A, B, C, D, E, F, G and I [4,6,7]. Most ABC transporters are located at the organelle membranes, and are involved in the transport of many substances, including hormones, heavy metal complexes, lipids, and glycosides in Arabidopsis [4].

The AtABCD1/PXA1/PED3/CTS1 is a peroxisome-localized ABC transporter and is involved in some metabolic and developmental processes including seed germination and seedling growth in Arabidopsis [2,4,8]. AtLACS6/7 and AtCGI-58 can physically interact with AtABCD1 to facilitate substrates into the β-oxidation pathway and to co-regulate lipid homeostasis and signaling [8,9]. Seed germination promoted by AtABCD1 could be induced by pectin degradation under the control of ABI5 [10].

In this study, we cloned a new ABC transporter gene, *GmABCA7* (GenBank accession: MH910628), from the soybean cultivar, Heinong 44, which encodes a protein of 954 amino acid residues. Currently, only AtABCA9 has been functionally identified from the A subfamily of Arabidopsis ABC transporters, which facilitates seed lipid synthesis at the endoplasmic reticulum [7,11]. GmABCA7 is localized at the peroxisome, suggesting GmABCA7 may have a different function compared to AtABCA9. The succinate and malate levels in the *GmABCA7*-overexpressing Arabidopsis seeds showed a marked increase compared to those in the wild type. Our data showed that the overexpression of *GmABCA7* may affect β-oxidation of fatty acids and promote seed germination in Arabidopsis, which provides a new strategy to cultivate high-vigorous seeds to increase crop yield.

## 2. Results

### 2.1. Structural Features of GmABCA7 and Its Gene Expression

**Figure 1:**
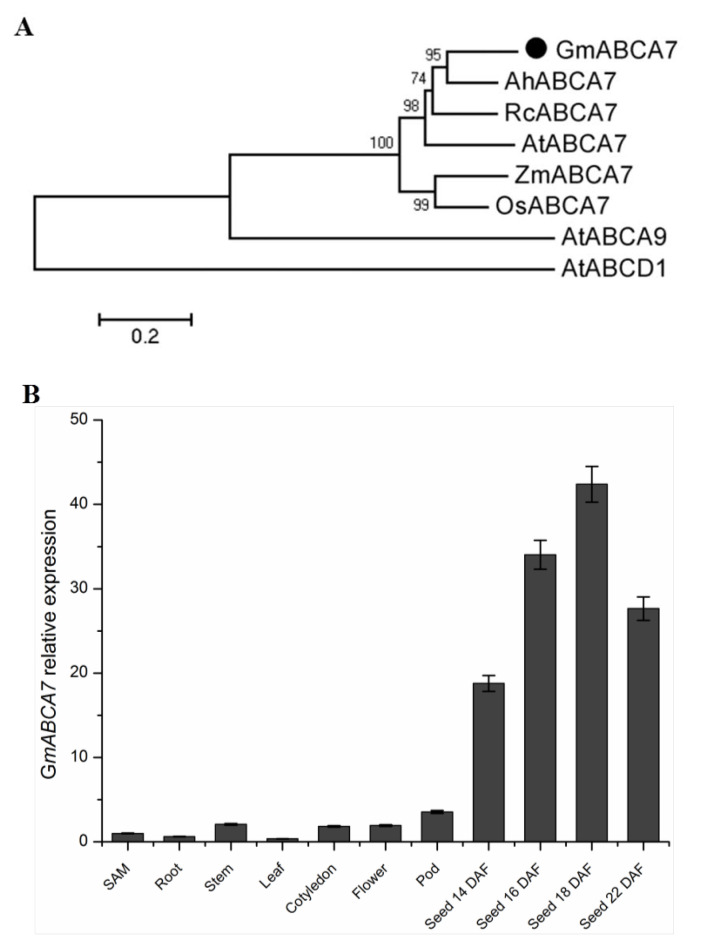
Phylogenetic analysis of GmABCA7 and its expression patterns in different tissues from *Glycine max*. (**A**) Phylogenetic tree analysis of GmABCA7 protein and other homologs from *Glycine max* (GmABCA7, MH910628); *Arabidopsis thaliana* (AtABCA7, NP_190362; AtABCA9, NP_200981; AtABCD1, NP_001328232); *Arachis hypogaea* (AhABCA7, XP_025701693); *Ricinus communis* (RcABCA7, XP_015579513); *Oryza sativa* (OsABCA7, XP_015651056) and *Zea mays* (ZmABCA7, XP_008652604). (**B**) Expression analysis of GmABCA7 in different soybean tissues by qRT-PCR. Roots, stems, SAMs and leaves from two-week seedlings, opening flowers and pods from adult plants, and seeds at 14, 16, 18 and 22 DAF were collected for analysis.

We cloned a new ABCA transporter gene from soybean, which encodes 954 amino acids (GenBank accession number: MH910628). A homology search against the Arabidopsis genome identified the highest homology with *AtABCA7*, thus the gene was named *GmABCA7* (Supplementary Figure S1). GmABCA7 had a 72.6%, 66.9%, 63.3%, 57.5% and 58.9% amino acid sequence identity to AhABCA7 (XP_025701693), RcABCA7 (XP_015579513), AtABCA7 (NP_190362), ZmABCA7 (XP_008652604) and OsABCA7 (XP_015651056), respectively (Figure 1A).

Quantitative RT-PCR (qRT-PCR) data showed that the *GmABCA7* was expressed in various tissues (SAM, root, stem, leaf, flower, cotyledon, pod and seeds at different developmental stages) of soybean, with the highest expression in seeds (Figure 1B). The gene was highly expressed between 14 and 22 DAF (Days after flowering) and peaked at 18 DAF in the developing seeds (Figure 1B). These results suggest that *GmABCA7* might be involved in the regulation of seed development in soybean.

### 2.2. GmABCA7 Is Localized at the Peroxisomes

**Figure 2:**
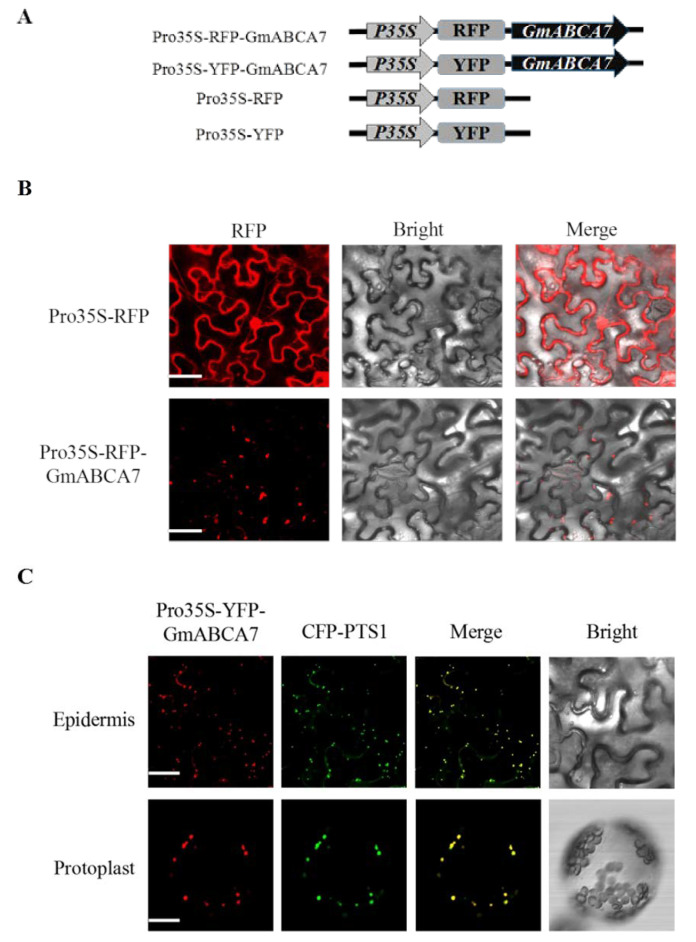
Subcellular localization of GmABCA7 in leaf epidermal cells of *Nicotiana benthamiana.* (**A**) Diagram of the Pro35S-RFP-GmABCA7, Pro35S-YFP-GmABCA7, Pro35S-RFP and Pro35S-YFP vectors. (**B**) Pro35S-RFP-GmABCA7 and Pro35S-RFP were expressed in tobacco epidermal cells respectively. Pro35S-RFP was used as a control. Bars = 50 µm. (**C**) Co-expression of Pro35S-YFP-GmABCA7 with CFP-PTS1. CFP-PTS1 was used as a marker for peroxisome. Protoplasts were from tobacco leaves co-expressing with CFP-PTS1 and YFP-GmABCA7. RFP, red fluorescent protein. YFP, yellow fluorescent protein. CFP, cyan fluorescent protein. Bars = 20 µm.

To investigate the function of the GmABCA7 transporter, the full-length GmABCA7 cDNA was ligated into the pCX-DR vector to generate the Pro35S-RFP-GmABCA7 and Pro35S-YFP-GmABCA7 vector (Figure 2A). We examined the subcellular localization of GmABCA7 by transiently expressing Pro35S-RFP-GmABCA7 in leaves of *Nicotiana benthamiana*. A punctate fluorescence pattern was observed in the tobacco leaf epidermal cells, which was completely different from the control (Figure 2B). The punctate fluorescence was similar to the identified peroxisome signals [12,13].

To further identify the subcellular localization of the GmABCA7 protein, we transiently co-expressed Pro35S-YFP-GmABCA7 with a CFP-PTS1-labeled peroxisomal marker (px-ck) [14] in tobacco leaves. Co-expression of the Pro35S-YFP-GmABCA7 and CFP-PTS1 clearly showed that the YFP and CFP signals completely overlapped in the leaf epidermal cells and leaf protoplasts (Figure 2C). These results suggest that GmABCA7 is localized at the peroxisomes.

### 2.3. Overexpression of GmABCA7 Promotes Seed Germination in Arabidopsis

**Figure 3:**
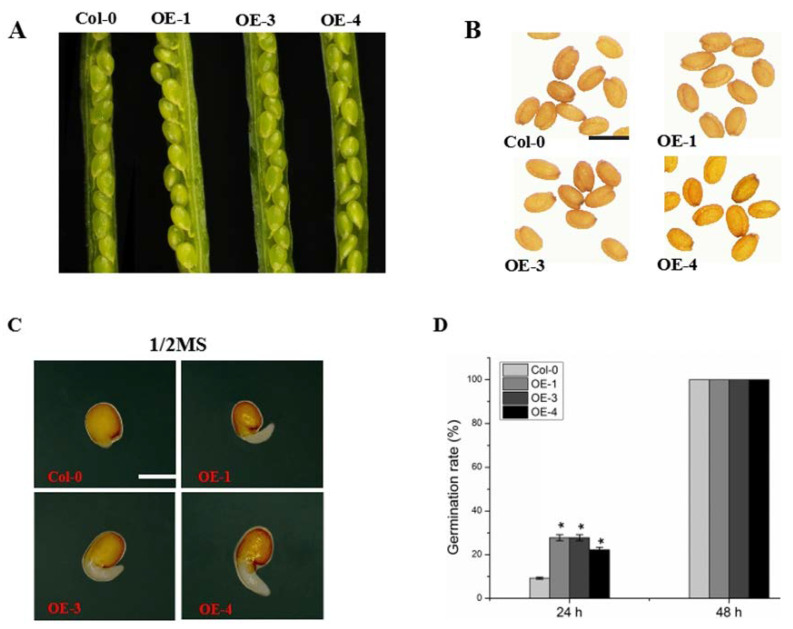
Overexpression of *GmABCA7* promoted seed germination in Arabidopsis. (**A**) Siliques at 12 days after flowering (12 DAF). (**B**) Seeds of the wild type and three transgenic lines (OE-1, OE-3 and OE-4). Scale bar = 500 µm. (**C**) Seed germination at 24 h after sowing on 1/2 MS medium. Scale bar = 0.5 mm. (**D**) Germination rates of wild type and *GmABCA7* transgenic seeds were measured 24 h and 48 h after sowing on 1/2 MS medium. The wild type and *GmABCA7* transgenic seeds (OE-1, OE-3 and OE-4) were grown with a photoperiod of 16 h, 22 °C/8 h, 20 °C (light/dark, respectively). Values are means ± SE of three independent experiments each using 18 seeds. Asterisks indicate a significant difference compared to the corresponding controls (* *p* < 0.05).

Three Arabidopsis transgenic lines (OE-1, OE-3 and OE-4) were examined for *GmABCA7* gene expression (Supplementary Figure S2A). To analyze the function of the GmABCA7 during seed development, we examined the transgenic lines in terms of seed-related traits. Developing seeds at 14 DAF and dry seeds from wild type and GmABCA7-overexpressing plants were observed under stereomicroscope. We found that there was no significant difference in seed size and 1000-seed weight between the transgenic lines and the wild type seeds (Figure 3A,B and Supplementary Figure S2B).

Lipid, sugar, starch and protein are the main storage substances in seeds. To determine whether overexpression of *GmABCA7* affects the seed storage substances, we examined the lipid, sugar, starch and protein content of the GmABCA7 transgenic lines (Supplementary Figure S2C–F). The overexpression of *GmABCA7* had no significant effects on the accumulation of the overall lipid, sugar and protein, except for increased C18:3 fatty acid contents in the Arabidopsis seeds.

To investigate the effect of overexpression *GmABCA7* on seed germination in Arabidopsis, the wild type and GmABCA7 transgenic seeds were grown on 1/2 MS medium, and the radicle protrusion was studied. We found that about 20% of seeds from the three transgenic lines had prominent radicles at 24 h after sowing, while much less in the wild type seeds; germination rates of the transgenic lines were 12.96% to 18.52% higher compared to the wild type (Figure 3C,D). All the seeds germinated at 48 h after sowing (Figure 3D). These results suggest that the overexpression of *GmABCA7* promoted seed germination in Arabidopsis.

The plant hormones abscisic acid (ABA) and gibberellin (GA) play important roles in the regulation of seed dormancy and the germination process. We studied the germination rates of seeds treated with exogenous ABA and GA. We found that germination rates of the transgenic seeds were higher than that of the wild type seeds upon 0.3 μM, 0.5 μM and 1.0 μM of the ABA treatments (Supplementary Figure S3A). Germination rates of the transgenic seeds were also significantly higher than that of the wild type upon 0.3 μM, 0.5 μM and 1.0 μM of the GA3 treatment (Supplementary Figure S3B). These results suggest that ABA and GA3 might not change the earlier germination phenotype of *GmABCA7*-transgenic seeds compared to wild type seeds.

### 2.4. Overexpression of GmABCA7 Increased the Levels of Gluconeogenic Pathway Precursors during Seed Germination

**Figure 4:**
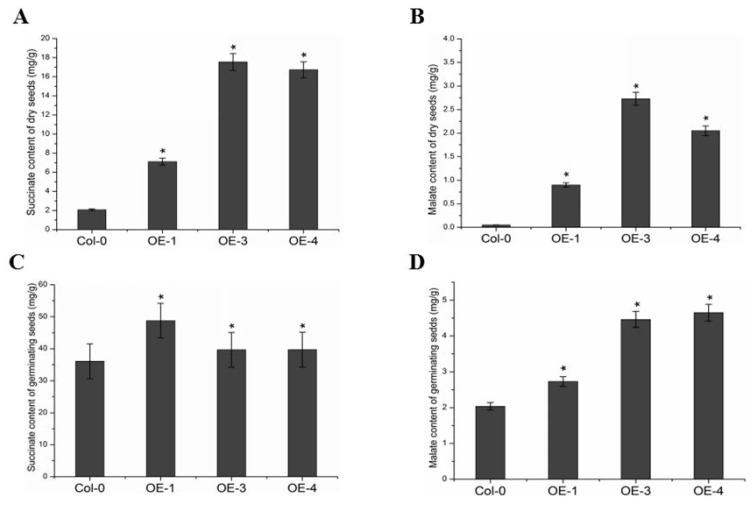
Succinate and malate levels in dry and germinating seeds. (**A**,**B**) Succinate and malate levels in mature dry seeds. (**C**,**D**) Succinate and malate levels in germinating seeds. Asterisks indicate a significant difference compared to the corresponding controls (* *p* < 0.05). Error bars indicate SEs from three replicates.

Fatty acids are metabolized by β-oxidation to acetyl-CoA [15], which is converted to succinate through a cycle of the glyoxylic acid [16]. Succinate moves into the mitochondrion and is converted to malate, which is then turned into sucrose through the gluconeogenic pathway, providing the carbon source and energy required for germination. To further investigate the function of *GmABCA7* during seed development and germination, we examined levels of the gluconeogenic pathway precursors. The levels of succinate and malate in mature dry seeds were significantly higher compared to the wild type (Figure 4A,B). These results suggest that the *GmABCA*7 had a positive impact on the β-oxidation process during seed development, thereby promoting the accumulation of succinate and malate in the seeds. Then, we soaked the seeds with distilled water at 37 °C for 72 h to initiate seed germination. The succinate and malate levels in the *GmABCA7* overexpression lines were significantly up-regulated (Figure 4C,D), probably due to the highly expressed *GmABCA*7 during germination in the transgenic lines (Supplementary Figure S4A).

As previously reported, β-oxidation could convert 2,4-dichlorophenoxybutyric acid (2,4-DB) into 2,4-dichlorophenoxyacetic acid (2,4-D), and the plants showed stunted root growth when treated with 2,4-DB [17]. *GmABCA*7 transgenic seedlings were more sensitive to 2,4-DB compared to the wild type (Supplementary Figure S5), suggesting GmABCA7 might enhance β-oxidation during germination.

These results suggest that the overexpression of *GmABCA7* might increase the levels of gluconeogenic pathway precursors via enhanced β-oxidation during seed germination in transgenic Arabidopsis.

## 3. Materials and Methods

### 3.1. Bioinformatic and Phylogenetic Analysis

The structure of the GmABCA7 protein was analyzed using an online website (http://smart.embl-heidelberg.de) on 2 July 2019 [18]. The relevant homologous protein sequences were downloaded from Phytozome (phytozome.jgi.doe.gov) on 2 July 2019 and NCBI (http://www.ncbi.nlm.nih.gov/Blast.cgi) on 2 July 2019, and were aligned using DNAMAN 8.0 (Lynnon Biosoft, San Ramon, CA, USA). The phylogenetic tree was constructed using the neighbor-joining (N-J) method provided by the MEGA software v. 5.0 [19].

### 3.2. Plant Materials and Growth Conditions

The soybean cultivar, Heinong 44, provided by the Institute of Genetics and Developmental Biology, Chinese Academy of Sciences, was grown at 25 °C with a photoperiod of 14 h/10 h (light/dark, respectively). The soybean root, stem, leaf, flower, pod, shoot apical meristem (SAM), cotyledon and seeds from different developmental stages (14,16,18 and 22 Days after flowering, DAF) were kept at −70 °C for subsequent RNA isolation.

*Arabidopsis thaliana* seeds of wild type (ecotype Col-0) and *GmABCA7* transgenic plants (OE-1, OE-3 and OE-4) were grown with a photoperiod of 16 h, 22 °C/8 h, 20 °C (light/dark, respectively). Seeds were harvested and stored at room temperature for about 2–4 weeks for the germination test.

### 3.3. RNA Isolation and Quantitative RT-PCR (qRT-PCR) Analysis

Total RNA was isolated from the soybean tissues and Arabidopsis transgenic siliques using BIOZOL Total RNA Extraction Reagent (Bioer Technology, Hangzhou, China) and the cDNA was synthesized using the cDNA synthesis kit (Takara, Japan), according to the manuals. For the qRT-PCR, the Hieff™ qPCR SYBR^®^ Green Master Mix (High Rox Plus) (Yeasen, Shanghai, China) and the Applied Biosystems StepOne system (Thermo Fisher Scientific, Waltham, MA USA) were used. The *GmCYP2.1* and *AtTIP41* genes were used as internal controls for the soybean and Arabidopsis, respectively [20,21]. Primers are listed in Supplementary Table S1.

### 3.4. Plasmid Construction and Plant Transformation

The coding sequence of *GmABCA7* (accession number: MH910628) was ligated into the plant binary vector pCX-DR and was under the control of the 35S CaMV promoter (GenBank: FJ905223) [22], resulting in the Pro35S-RFP-GmABCA7 vector. The *Kpn*I-35S-YFP-*Kpn*I fragment was PCR amplified from PXYK (YFP-PTS1) using the Q5 enzyme (NEB, Beijing). Subsequently, the *Kpn*I-35S-DsRED-*Kpn*I fragments of both the Pro35S-RFP-GmABCA7 and pCX-DR were replaced by *Kpn*I-35S-YFP-*Kpn*I, to generate Pro35S-YFP-GmABCA7 and Pro35S-YFP, respectively. The *Kpn*I-35S-YFP-*Kpn*I regions of these two vectors were confirmed by sequencing.

Constructs were introduced into an *Agrobacterium tumefaciens* strain GV3101 by electroporation, and the Arabidopsis plants transformed by floral dipping [23]. The Pro35S-YFP-GmABCA7 transgenic T1 plants were selected on a half-strength MS medium with 40 mg/L of hygromycin and 200 mg/L of cefotaxime, and further confirmed by PCR. The seeds from the transgenic T1 plants were grown for two more generations to screen for homozygous transgenic lines. The primers used are listed in Supplementary Table S1.

### 3.5. Subcellular Localization Analysis

Each plasmid was introduced into the *Agrobacterium tumefaciens* GV3101. All Agrobacterium strains were cultured in 5 mL of LB liquid medium overnight, and then the cells were collected by centrifugation at 4000 rpm, 4 °C for 10 min, and was re-suspended in an infiltration buffer (100 μM acetosyringone, 10 mM MES-KOH and 10 mM MgCl_2_) to an OD600 of 0.6 to 0.8 [24]. The bacteria were infiltrated into tobacco leaves and the leaves were kept for 1 day in the dark, then subsequently under normal lighting (16 h, 25 °C/8 h, 22 °C, light/dark, respectively) conditions for 2 days. Enzymatic hydrolysis was used to free tobacco mesophyll protoplasts [25], prior to the observation of the GmABCA7 protein subcellular localization using a confocal microscope (Olympus FV1200, Japan). Agrobacterium strains harboring pCX-DR and px-ck (CFP-PTS1, http://www.bio.utk.edu/cellbiol/markers/, accessed on 8 March 2019) were used as the control and maker, respectively [14].

### 3.6. Seed Lipid, Fatty Acid, Protein and Sugar Analysis

The seed lipid was quantified according to the hexane extraction method as described [26]. An amount of 100 mg of dry seeds was ground into hexane, then, the hexane supernatant was collected after being centrifuged at 20,000 rpm for 5 min. The seed lipid content was calculated by the amount of lipid after hexane evaporation.

An amount of 50 mg of seeds was used for the extraction of fatty acids as described [27]. After extraction, the fatty acids were subjected to gas chromatography (GC-2010, Shimadzu).

Seed protein content was determined using an automated Kjeldahl method [28]. The ground sample (100 mg) was digested by the addition of 10 mL sulfuric acid and 3.0 g of a CuSO4–K_2_SO4 mixture (g/g, 1:10). The nitrogen amount (%) was calculated by using Kjeldahl K-1100 (Hanon) and then converted to the protein amount (%) by multiplication by 6.25.

An amount of 50 mg of seeds was used for the extraction of sugar as described [27]. The ground seeds were extracted with 80% (*v*/*v*) ethanol at 80 °C for 40 min. After extraction, the supernatant was used to determine the levels of glucose, sucrose and fructose. The samples after the soluble sugar extraction were used for starch quantification.

### 3.7. Succinate and Malate Analysis

Succinate and malate were extracted from the samples by a variation of the hot water extraction method [29], and the extract was subjected to high performance liquid chromatography analysis (HPLC, Agilent 1100). Ground samples (0.1 g) were mixed with 1 mL of deionized water and boiled in a 100 °C water bath for 1 h. The filtrate was centrifuged (10,000 rpm for 15 min at 25 °C) and transferred to a new centrifuge tube. Finally, it was filtered through a 0.45 mm membrane filter prior to the HPLC analysis.

### 3.8. Statistical Analysis

Statistical analysis was performed using Microsoft Excel, and analysis of variance was performed using the SPSS 9.0 program. IBM SPSS Statistics 22 was used for the significance analysis. Different letters represented significant differences (*p* < 0.05). Origin 8.5 was used to map the results.

## 4. Discussion

Oilseed’s germination depends on the breakdown of storage lipids. The fatty acids are oxidized in one class of peroxisomes called a glyoxysome. Plant peroxisomes participate in many metabolic and signaling pathways, such as the generation of signaling molecules, detoxification, and responses to abiotic and biotic stresses [30]. The subfamily D of ABC transporters are believed to be required for these substrates to pass through the peroxisome membrane, but the transport mechanism is still unclear.

Up to now, few peroxisomal transporters have been identified in plants. The function of the human peroxisomal ABC transporters (HsABCD1, HsABCD2 and HsABCD3) was found to transport fatty acyl-CoA [31,32]. Arabidopsis AtABCD1 is the most studied plant peroxisomal ABC transporter and has been identified for multiple functions, including the transport of the fatty acids, indole-3-butyric acid (IBA) and 12-oxophytodienoic acid (OPDA), into peroxisomes for β-oxidation [9,31,32,33,34,35]. In this study, co-expression of YFP-GmABCA7 with CFP-PTS1 showed that GmABCA7 was localized at the peroxisomes (Figure 2), and that *GmABCA7* was highly expressed in developing soybean seeds (Figure 1). These results suggest that GmABCA7 might regulate the transport of certain substances for β-oxidation in seeds.

Sugar, starch and lipid metabolism play an important role in plant seed germination. We found that the overexpression of *GmABCA7* only affected the seed fructose level in OE-3 and OE-4 but did not significantly change the overall levels of total soluble sugar, starch, lipids and proteins (Supplementary Figure S2C–F). Furthermore, we found that there was no difference in seed size and 1000-seed weight between the *GmABCA7*-overexpressing lines and wild type (Figure 3A,B and Supplementary Figure S2B). These results suggest that the earlier germination of the transgenic seeds is not due to the accumulation of carbon sources.

Succinate and malate are important gluconeogenic pathway precursors. In this study, we found that *GmABCA7*-overexpressing lines significantly enhanced succinate and malate levels compared to the wild type, which suggest that the overexpression of *GmABCA7* affected the β-oxidation during seed germination in transgenic Arabidopsis seeds (Figure 4). *GmABCA7* was highly expressed in seeds after imbibition in water, while the *AtABCA7* expression did not change much in the wild type seeds, suggesting the *GmABCA7* functioned at the germination stages in the transgenic lines (Supplementary Figure S4). Furthermore, *GmABCA7* transgenic seedlings were more sensitive to 2,4-DB (Supplementary Figure S5). It is possible that GmABCA7 facilitates the β-oxidation process during seed development, thereby promoting the accumulation of succinate and malate in seeds.

In conclusion, the overexpression of *GmABCA7* might affect β-oxidation in seeds, thus providing more carbon source and energy for seed germination. When *GmABCA7* is overexpressed in Arabidopsis, the endogenous *AtABCA7* expression might also affect the validation of the GmABCA7 function. Therefore, the function of GmABCA7 needs to be further verified in soybean. Up to now, more and more peroxisome localized proteins such as AtABCD1 and AtCGI-58 have been characterized [2,9]. Our study identified a new ABCA transporter from soybean, which provides a promising target to increase seed vigor and seed germination for soybean breeding.

## Data Availability

All the data in this study are included in this published article and its Supplementary Material files.

## Supplementary Materials

The following supporting information can be downloaded at: https://www.mdpi.com/article/10.3390/ijms23042389/s1.
