# Supplementary material for: Overexpression of Peroxisome-Localized GmABCA7 Promotes Seed Germination in Arabidopsis thaliana
Source: Int J Mol Sci. 2022 Feb 21;23(4):2389. doi: 10.3390/ijms23042389 (PMC8879324; doi:10.3390/ijms23042389)
Supplement: Supplementary file 1 [file ijms-23-02389-s001.zip › ijms-1559221-supplementary.pdf]

International Journal of
Molecular Sciences

MDPI

Table S1. The list of primers used in PCR reactions.

Primer name	Primer sequences (5'→3')
GmCYP2.1-RT-F	CGGGACCAGTGTGCTTCTTCA
GmCYP2.1-RT-R	CCCCTCCACTACAAAGGCTCG
AtTIP41-RT-F	CTGGCTGACAATGGAGTGTCACTTCTG
AtTIP41-RT-R	GAACAGTTGGTGCCTCATCTTCGC
GmABCA7-RT-1F	GGCAGACTTAAAAATCTTAAAGGCTCAC
GmABCA7-RT-1R	GCACGCTTGACAACATTCCATAAGC
GmABCA7-RT-2F	CAT TCC ATG GAA GAG GCA GAA GCC
GmABCA7-RT-2R	CAT TTG GGG TGA GTT TCT GCA CCA TG
GmABCA7-F	T ATGGCAAATCCTGTTCCCGCGA
GmABCA7 -R	TTATGACAAGGTGTCAAATGCCT
YFP-*KpnI*-FP	GGGGTACCAGCACGACACACTTGTCTAC
YFP-*KpnI*-RP	GGGGTACCCTGTACAGCTCGTCCATGC

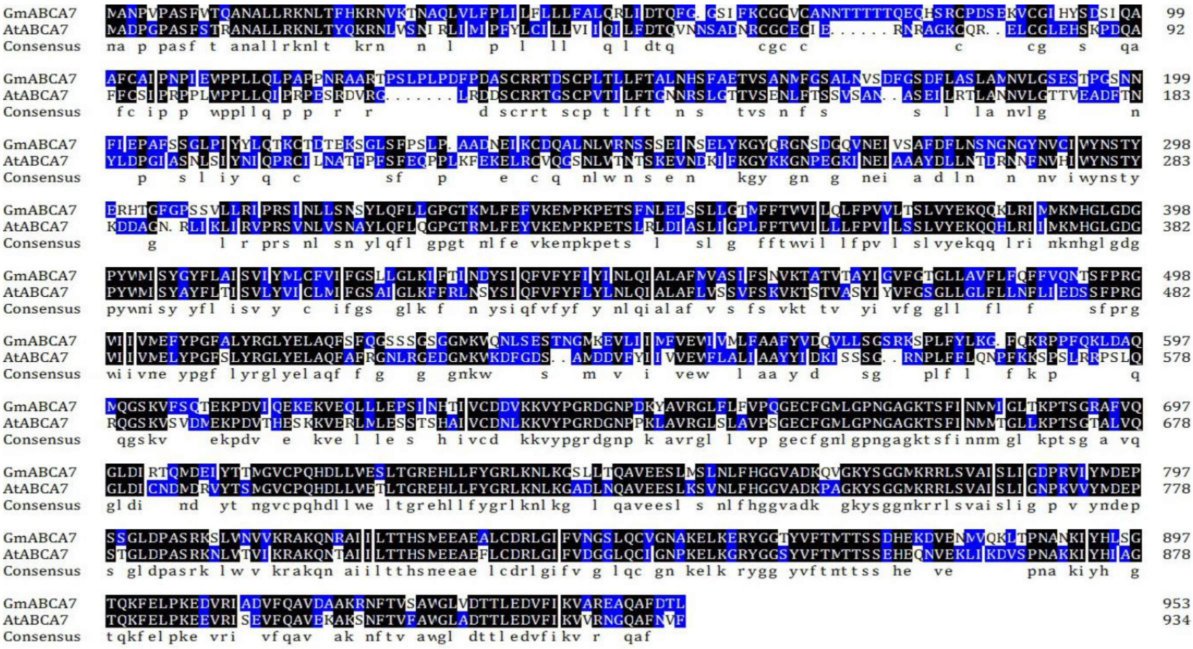

Figure S1. Amino acid sequence alignment of ABCA7 from soybean and Arabidopsis.

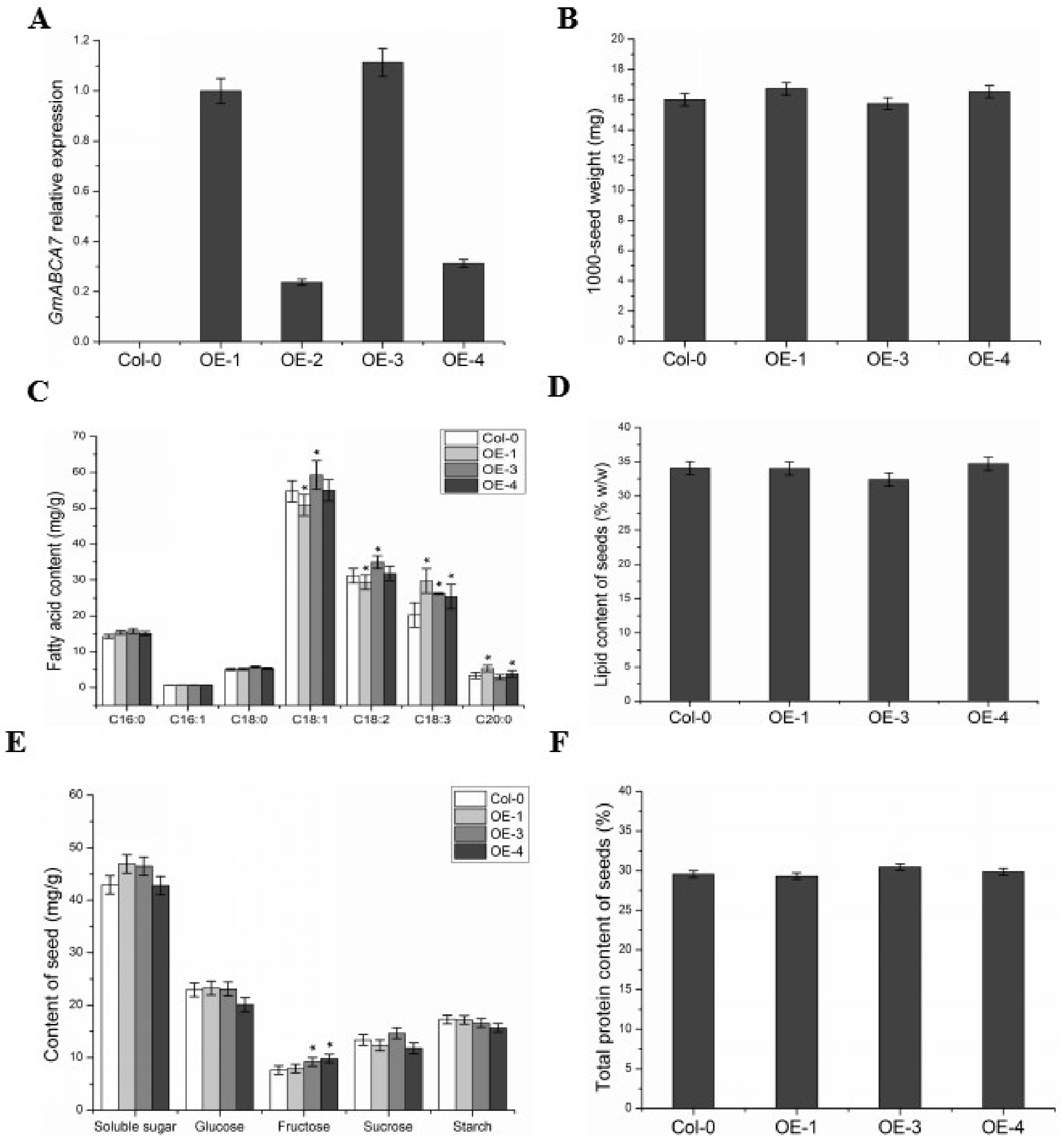

Figure S2. Analysis of seed phenotype of wild type (Col-0) and *GmABCA7* transgenic plants. (A) Expression of *GmABCA7* in transgenic plants. The relative expression level of *GmABCA7* was normalized using *AtTIP41* as an internal control. (B) The 1000-seed weight of wild type and *GmABCA7* transgenic plants. 1000 seeds from different lines were used for weighing. (C) Major fatty acid levels in dry seeds of wild type and three transgenic lines (OE-1, OE-3 and OE-4). (D) Total lipid levels in wild type and transgenic seeds. (E) Levels of glucose, fructose, sucrose and starch in dry seeds of wild type and *GmABCA7* transgenic lines. (F) Total protein level in seeds of wild type and *GmABCA7* transgenic plants. Values are means ± SE of three independent measurements.

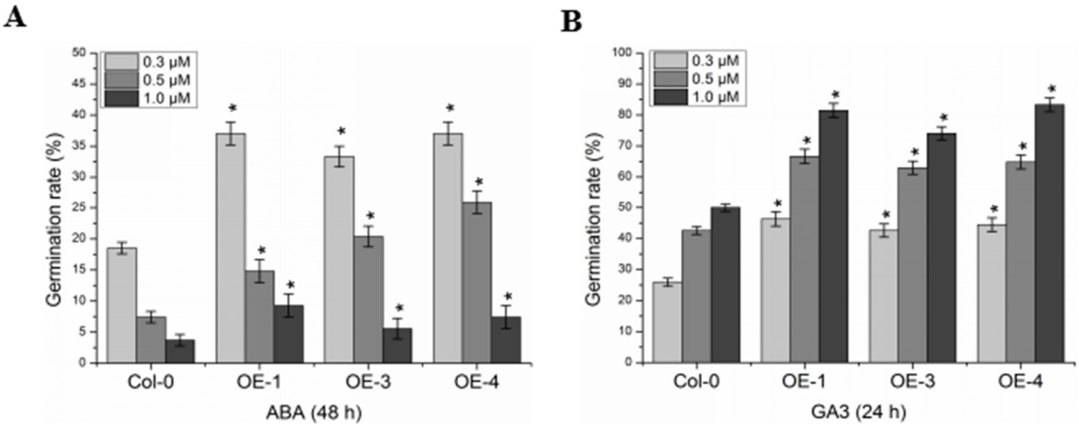

Figure S3. Seed germination rates upon ABA and GA3 treatment. (A) Seed germination rates upon treatment with various concentrations of ABA (0.3 μM, 0.5 μM and 1.0 μM). Germination rates were measured 48 h after seeds were sown. (B) Seed germination rates upon treatment with various concentrations of GA3 (0.3 μM, 0.5 μM and 1.0 μM). Germination rates were measured 24 h after seeds were sown. Wild type and GmABCA7 transgenic seeds (OE-1, OE-3 and OE-4) were grown with a photoperiod of 16 h, 22°C /8 h, 20°C (light/dark). Values are means ± SE of three independent experiments each using 18 seeds. Asterisks indicate a significant difference compared to the corresponding controls (*P <0.05).

A

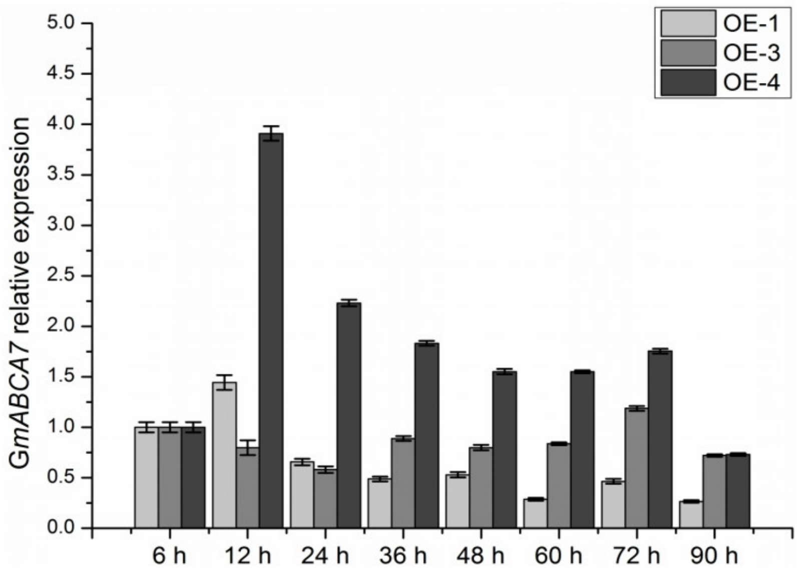

B

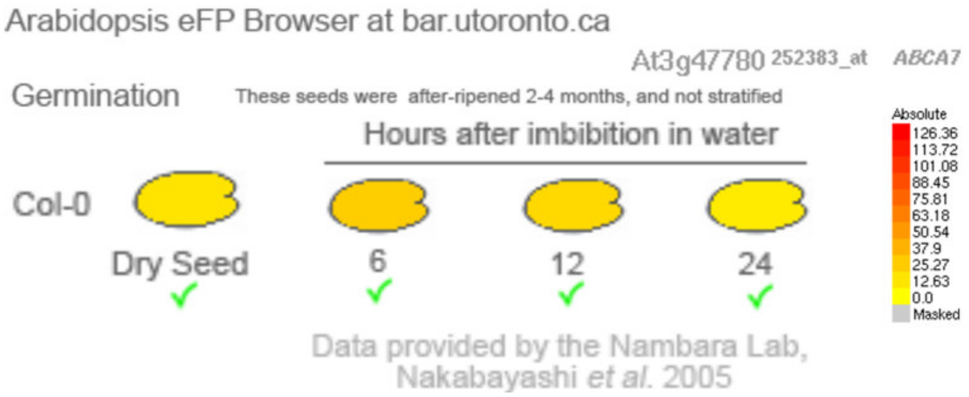

Figure S4. Relative expression levels of *GmABCA7* and *AtABCA7* in seeds after imbibition in water. (A) *GmABCA7* expression levels in transgenic seeds. Seeds were imbibed in purified water at 37°C for 6 h, 12 h, 24 h, 48 h, 60 h, 72 h and 90 h prior to quantification of *GmABCA7* expression levels. Values are the means ± SE of three independent measurements. (B) *AtABCA7* (AT3G47780) expression levels in wild type seeds. Data were obtained from the website: http://bar.utoronto.ca/efp_arabidopsis/cgi-bin/efpWeb.cgi

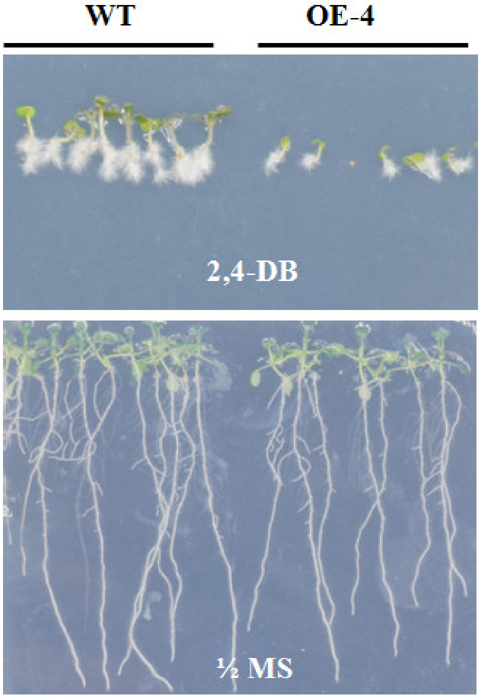

Figure S5. Response of wild type (WT, Col-0) and *GmABCA7* transgenic seedlings (OE-4) to 2,4-DB. Seedlings were grown vertically on 1/2 MS plates with or without 0.7 μM 2,4-DB for 9 days in the presence 1% (w/v) sucrose.
